# Alternative miRNAs? Human sequences misidentified as plant miRNAs in plant studies and in human plasma

**DOI:** 10.12688/f1000research.14060.1

**Published:** 2018-02-28

**Authors:** Kenneth W. Witwer

**Affiliations:** 1 Department of Molecular and Comparative Pathobiology, Johns Hopkins University School of Medicine, Baltimore, MD, 21205, USA; 2 Department of Neurology, Johns Hopkins University School of Medicine, Baltimore, MD, 21205, USA

**Keywords:** microRNA, xenomiR, diet, RNAi, biofluid, plasma

## Abstract

**Background:** A 2017 study reported that “Plant miRNAs found in human circulating system provide evidences of cross kingdom RNAi”. Analysis of two human blood plasma sequencing datasets was said to provide evidence for uptake of plant miRNAs into human plasma. The results were also purportedly inconsistent with contamination.

**Methods:** Sequences from public datasets and miRNA databases were compared with results downloaded from the website of the reporting journal.

**Results:** Only one putative plant miRNA (“peu-MIR2910) mapped consistently above background, and this sequence is found with 100% identity in a human rRNA. Several other rarer but consistently mapped putative plant miRNAs also have 100% or near 100% matches to human transcripts or genomic sequences, and some do not appear to map to plant genomes at all.

**Conclusions:** Reanalysis of public data suggests that dietary plant xenomiR uptake is not supported, but instead confirms previous findings that detection of rare plant miRNAs in mammalian sequencing datasets is artifactual. Some putative plant miRNAs, including MIR2910 and MIR2911, may represent human sequence contamination or other artifacts in plant studies, emphasizing the need for rigorous controls and data filtering strategies when assessing possible xenomiRNAs.

## Introduction

Reports of plant or other dietary miRNAs, or xenomiRs, entering mammalian circulation through the diet ^1– 4^ generated initial excitement for the xenomiR transfer hypothesis, yet negative results of replication and reproduction studies have cast doubt on xenomiR transfer as a general mechanism ^5– 11^. A prominent claim of xenomiR function ^1^ has also failed rigorous reproduction ^7^, unmasked as the result of an uncontrolled variable in the original experiment. Analyses of public datasets have revealed that studies of xenomiRs and other foreign-origin nucleic acids are fraught with artifacts: combinations of contamination, amplification or sequencing errors, permissive analysis pathways, and batch effects ^8, 10, 12– 16^. A particularly comprehensive study recently found that foreign miRNAs in human biofluids and tissues do not match human food consumption, are marked by batch effects, and are thus most parsimoniously explained as artifacts ^13^. Studies of organisms with no exposure to plants have also found evidence of the same types of apparent plant contamination that plague some measurements of human samples ^8, 17^. Liu *et al.* ^18^ mapped sequencing data from two studies of human plasma and other samples ^19, 20^ to various plant genomes using a 2010 plant miRNA database, PMRD ^21^, concluding that previous reports of dietary xenomiR transfer are supported. In this brief report, these results are examined critically.

## Methods

Plant mapping results from Liu *et al.* ^18^ (total mapped counts) were downloaded from the BMC Genomics website. Accession numbers of sequencing datasets were checked against the publications of Ninomiya *et al.* ^19^ and Yuan *et al.* ^20^, as well as the Sequence Read Archive ( SRA). Data were sorted and analyzed in Microsoft Excel for Mac 2011, Version 14.7.1. Plant miRNA sequences were obtained from miRBase ^22^. Because certain plant sequences have been removed from miRBase because they have been identified as ncRNA degradation artifacts, the plant microRNA database ( PMRD) ^21^ was consulted; however, repeated attempts to access the site were unsuccessful, so information was retrieved instead from miRMaid ^23^ or miRNEST 2.0 ^24^. Supplementary File 1 contains the relevant count data from the Ninomiya *et al.* ^19^ and Yuan *et al.* ^20^ studies.

An earlier version of this article can be found on bioRxiv ( https://doi.org/10.1101/120634).

## Results

### Data evaluation

A cross-check of the source files and articles shows that the plasma data evaluated by Liu *et al.* were from 198 plasma samples, not 410 as reported. Ninomiya *et al.* sequenced six human plasma samples, six PBMC samples, and 11 cultured cell lines ^19^. Yuan *et al.* sequenced 192 human plasma libraries (prepared from polymer-precipitated plasma particles) ^20^. Each library was sequenced once, and then a second time to increase total reads. Counts were presented as reads per million mapped reads (rpm) ^20^. In contrast, Liu *et al.* appear to have reported total mapped reads in their data table ^18^. Yuan *et al.* also set an expression cutoff of 32 rpm (log2 rpm of 5 or above). With an average 12.5 million reads per sample (the sum of the two runs per library), and, on average, about half of the sequences mapped, the 32 rpm cutoff would translate to around 200 total reads in the average sample as mapped by Liu *et al.* ^18^.

### Only one putative plant miRNA above background levels

Consulting the Liu *et al.* mapping table ^18^ and the SRA, results from duplicate sequencing runs from the Yuan *et al.* dataset were combined, and two samples without reliable replicates were eliminated. A total of 1294 putative plant miRNAs had at least one mapped read in at least one of the remaining 190 samples. However, many of these miRNAs were identical orthologs or paralogs, and most were mapped at one or fewer rpm on average, and in only a small minority of samples. Across all samples, only one putative plant miRNA mapped above a median 200 read cutoff, roughly corresponding to the 32 rpm cutoff of Yuan *et al.* ( Table 1). All other RNAs, including previously reported xenomiRs such as MIR159a, MIR168a, and the plant ribosomal degradation fragment MIR2911 ^25– 27^, were thus below the level of background noise established by the original investigators. Indeed, previously reported xenomiRs were mapped in few samples and below 1 rpm. The absence of these RNAs is confirmed by Liu *et al.*’s analysis of the Ninomiya, *et al.* study ^19^, where MIR159a, MIR168a, and MIR2911 mapped in none of the plasma samples. The single putative plant miRNA that mapped above background levels in this study was, again, peu-MIR2910 ( Table 2).

**Table 1.** Summary of the most frequently mapping putative plant miRNAs in the Liu *et al.* analysis of datasets from Yuan *et al.*. Here, data from only 190 of 192 plasma samples were included, since all but the excluded 2 samples were successfully sequenced twice. miRNA inclusion criteria were: 1) Three total mapped reads according to Liu *et al.*’s data in at least 10% of the samples and 2) discoverable putative mature sequence through miRBase, miRMaid, or miRNEST 2.0. An “estimated median rpm” value was calculated based on median total counts, average reads, and the midoint of the reported mapping percentage range. miRNAs with perfect human matches are in red. Note that only MIR2910 consistently exceeds the rpm threshold set by Yuan *et al.*.

Putative miRNA	Samples w/ reads ≥3	Average total counts	Median total counts	Max	Est. median rpm in avg sample
**peu-MIR2910**	190	1143.4	1072.5	2020	180.6
**peu-MIR2916**	190	119.7	115	315	19.4
**tae-MIR2005**	178	8.7	9	23	1.5
**peu-MIR2914**	169	14.3	9	348	1.5
**tae-MIR2018**	167	5.9	6	14	1.0
**ath-MIRf10482-akr**	161	7.3	6	29	1.0
**ppt-MIR896**	147	4.6	4	15	0.7
**ptc-MIRf12412-akr**	47	2.7	2	18	0.3
**peu-MIR2911**	42	2.6	2	10	0.3
**ppt-MIR894**	39	2.3	2	18	0.3
**ptc-MIRf12524-akr**	28	1.8	1	5	0.2

**Table 2.** Putative plant miRNA mapping from the Ninomiya *et al.* dataset. This dataset consisted of both cellular and plasma miRNA. Here, all results are shown in the left half of the table, and plasma results on the right. miRNA inclusion criteria were: 1) Three total mapped reads according to Liu *et al.*’s data in at least 10% of the samples (cells and plasma together) and 2) discoverable putative mature sequence through miRBase, miRMaid, or miRNEST 2.0. “Avg rpm” is calculated from the total mapped reads and total reads per sample (not mapped reads). Putative miRNAs that met inclusion criteria in the Yuan *et al.* study are italicized, and sequences with perfect human matches are in red.

	Cells (n=17) and plasma (n=6)					Plasma only (n=6)				
Putative miRNA	Samples w/reads ≥3	Average	Median	Max	avg rpm	Samples w/ reads ≥3	Average	Median	Max	avg rpm
***peu-MIR2910***	21	370	41.5	5369	21.3	6	1210	480.5	5369	83.2
**ptc-MIRf10804-akr**	17	26	26	60	1.2	0			0	0
***ptc-MIRf12412-akr***	17	63	61	156	2.8	0			0	0
***tae-MIR2018***	17	39	29	163	1.7	0			0	0
***ptc-MIRf12524-akr***	10	13	10.5	31	0.4	2	16	16	31	0.4
***tae-MIR2005***	7	17	11	69	0.4	0			0	0
**ath-MIRf10045-akr**	4	5	4	10	0.1	0			0	0
**ath-MIRf10046-akr**	4	5	4	10	0.1	0			0	0
***peu-MIR2914***	3	26	11	81	0.3	3	33.7	19	81	1.2
**peu-MIR2915**	3	5	4.5	8	0.0	0			0	0

### Lowering the threshold: still only a handful of possible xenomiRs

Since only one plant miRNA appeared to map consistently above background, the inclusion threshold of Yuan *et al.* was relaxed to include all miRNAs with three or more mapped reads (Liu *et al.* data) in 10% or more of the samples from either study. These are rather permissive criteria but may at least screen out some false positives due to amplification and sequencing errors. All samples from the Ninomiya study were included, despite the fact that most were not plasma. 11 miRNAs satisfied these criteria for the Yuan *et al.* data ( Table 1). (One low-mapping miRNA was excluded because its sequence could not be found in miRBase ^22, 28^, miRMaid ^23^, miRNEST 2.0 ^24^ or indeed through any searches attempted.) 10 satisfied the criteria from the Ninomiya study ( Table 2), including one sequence that was part of another (compare ath-MIRf10046-akr and ath-MIRf10045-ak, Table 3). However, if only the plasma samples from the latter study are considered, three miRNAs remain ( Table 2). In total, 15 putative miRNAs satisfied the permissive inclusion criteria, including five (Yuan only), four (Ninomiya only), and six (both) ( Table 3).

**Table 3.** Putative plant miRNAs mapped by Liu *et al.* from the Yuan *et al.* or Ninomiya *et al.* studies. Inclusion criteria were: 1) Three total mapped reads according to Liu *et al.*’s data in at least 10% of the samples in the respective studies and 2) discoverable putative mature sequence through miRBase, miRMaid, or miRNEST 2.0. miRNA status was considered unlikely if miRBase listed the miRNA as a non-miRNA or if the sequence mapped to non-miRNA regions. Human matches were exact (with an example given), “partial” (at least 15 nt stretches with 100% identity), or as otherwise described. Note that the ath-MIRf10046-akr is found within the ath-MIRf10045-akr sequence.

Putative miRNA	Yuan *et al.* or Ninomiya *et al.*	Sequence	miRBase	miRNA status in plant	Human matches?	Example match
**peu-MIR2910**	Both	UAGUUGGUGGAGCGAUUUGUC	No	rRNA fragment	18s rRNA	NR_003286.2
**peu-MIR2916**	Yuan	UGGGGACUCGAAGACGAUCAUAU	Yes	Possible, but 20 nt maps to rRNA	Partial	
**tae-MIR2005**	Both	GGGUGUAUAGCUCAGUUGG	No	Unlikely: plant mitochondrial genome	Partial	
**peu-MIR2914**	Both	CAUGGUGGUGACGGGUGACGGAG	No	rRNA fragment	Partial	
**tae-MIR2018**	Both	GCCCGUCUAGCUCAGUUGGU	No	Unlikely; maps to many plant transcripts	Partial	
**ath-MIRf10482-akr**	Yuan	UCUACUCGACUAGGUGGUCGAGUGG	No	Maps to *Arabidopsis*	Partial	
**ppt-MIR896**	Yuan	GUCAAUUUGGCCGAGUGGUUAAGGC	No	tRNA fragment	Partial	
**ptc-MIRf12412-akr**	Both	GCUGGGAUUACAGGCGUGAGCCACC	No	Does not map to *Populus*	Many exact	XR_001736898.1
**peu-MIR2911**	Yuan	GGCCGGGGGACGGGCUGGGA	No	rRNA fragment	1 mismatch in a 20-nt stretch	NC_018921.2
**ppt-MIR894**	Yuan	CGUUUCACGUCGGGUUCACC	Yes	Yes, but other transcriptome matches	No	
**ptc-MIRf12524-akr**	Both	CCUGUAAUCCCAGCUACUCGGG	No	Does not map to *Populus*	Many exact	NG_053018.1
**ath-MIRf10045-akr**	Ninomiya	UCUACUCGACCUGGUGGUCGAGUGGU	No	Unlikely; chromosomal region	Partial	
**ath-MIRf10046-akr**	Ninomiya	CUCGACCUGGUGGUCGAGUGGU	No	Part of above sequence	Partial	
**peu-MIR2915**	Ninomiya	CCCGUCUAGCUCAGUUGGUA	No	tRNA fragment; sequence found in many transcripts	Partial	
**ptc-MIRf10804-akr**	Ninomiya	CCUGUAAUCCCAGCACUUUGG	No	Unlikely; microsatellite sequence	Many exact, also hsa-miR- 3929 precursor (1 mismatch)	XR_001736898.1 (antisense)

### To miR or not to miR

As miRNA discovery, validation, and annotation has advanced, numerous reported miRNAs have been reclassified as degradation fragments of other noncoding RNAs (ncRNAs). A classic example is MIR2911, a plant rRNA degradation fragment that has been misidentified as a microRNA. Interestingly, only 2 of the 15 miRNAs identified as plant miRNAs in this study are annotated in miRBase. Although some of these sequences may represent rare or unusually structured miRNAs, several are part of non-miRNA ncRNAs or other sequences that seem unlikely, at least at first glance, to give rise to microRNAs. Among the apparently misidentified miRNAs is MIR2910, the most abundant plant miRNA identified by Liu *et al.*. The MIR2910 sequence, UAGUUGGUGGAGCGAUUUGUC, is found in the highly conserved and expressed large subunit (LSU) rRNA of plants, and has been specifically removed from miRBase as a non-miRNA. Even the two identified miRNAs that remain in miRBase, MIR2916 and MIR894, are not above question. A 20 nucleotide stretch of MIR2916 map to rRNA, while the full MIR894 sequence appears to be found in a variety of plant transcripts.

### Human sequences in the plant database and vice-versa

Curiously, several sequences did not map to the species to which they were ascribed by the PMRD ^21^. Unfortunately, the PMRD could not be accessed directly during this study; however, other databases appear to provide access to its contents. Specifically, ptc-MIRf12412-akr and ptc-MIRf12524-akr did not map to *Populus* or to other plants. The poplar tree is also not a common dietary staple of human populations. In contrast, both sequences mapped with 100% identity and coverage to numerous human sequences ( Table 3). ptc-MIRf10804-akr had numerous 100% identity human matches, plus a 1-mismatch alignment to the human miR-3929 precursor. Other miRNAs, including MIR2911, also displayed some lesser degree of matching to human transcripts or the genome. Strikingly, the putative MIR2910 sequence is not only a fragment of plant rRNA; it has a 100% coverage, 100% identity match in the human 18S rRNA (see NR_003286.2 in GenBank; Table 3). These matches of putative plant RNAs with human sequences are difficult to reconcile with the statement of Liu *et al.* that BLAST of putative plant miRNAs "resulted in zero alignment hit" ^18^, suggesting that perhaps a mistake was made, and that the BLAST procedure was performed incorrectly.

## Discussion

In mammalian studies, mapping of MIR2910 and other dubious plant miRNAs is best explained as mapping of human degradome fragments to plant RNAs that are in some cases genuine sequences but not miRNAs, and in other cases, human sequences that have contaminated plant RNA samples and databases. Re-analysis of the results of Liu *et al.* ^18^ thus echoes the recent findings of Kang, Bang-Berthelsen, and colleagues ^13^, as well as previous negative findings surrounding dietary xenomiRs, summarized above. A stringent data analysis procedure, such as filtering all reads against the ingesting organism genome/transcriptome with one or two mismatches, then requiring perfect matches of remaining reads against plant or other foreign organisms, would engender higher confidence that “foreign” RNAs are not simply amplification or sequencing artifacts. Indeed, pre-mapping to the ingesting organism’s genome may not be sufficient; as shown ^13^, the largest number of xenomiRs in some human studies are from rodents, likely because of proximity in research laboratories. Therefore, it may be best to screen against mammalian sequences in general, and perhaps also against widespread microbe contaminants. Of course, even the most stringent analysis procedures cannot distinguish a physical contaminant from a “real” read; therefore strict process controls are also needed to assess possible contamination. In general, such controls have not been done in existing studies.

This report underlines the danger in assuming that xenomiRs in mammalian material originate from the diet. When the species and roles are reversed—for example, with the finding of human sequences in a list of poplar tree miRNAs—few analysts would conclude that poplar trees consume humans. The simplest explanation is that the sequenced plant material was contaminated with human nucleic acid. In the same way, the extremely low-level, variable, and batch-effect prone concentrations of several plant sequences in human plasma and tissue could be due to uptake from the diet, albeit at levels far too low to affect physiologic processes. However, artifact remains the simplest explanation.

## Data availability

Data used in these analyses were downloaded from the supplementary materials of Liu *et al.* ^18^ and are summarized in Supplementary File 1. Source data for the Ninomiya, *et al.* study ^19^ are under DRA002550, DRX021324–DRX021346, and DRR023270–DRR023292 ( DNA Data Bank of Japan), and, for the Yuan, *et al.* study ^20^, available as GSE71008 ( Gene Expression Omnibus).

## Supplementary material

**Supplementary File 1:** Tabs for the miRNA counts from the Yuan *et al.* and Ninomiya *et al.* datasets.

## References

[1] Zhang L Hou D Chen X: Exogenous plant MIR168a specifically targets mammalian LDLRAP1: evidence of cross-kingdom regulation by microRNA. *Cell Res.* 2012;22(1):107–126. 10.1038/cr.2011.158 21931358 PMC3351925

[2] Chin AR Fong MY Somlo G: Cross-kingdom inhibition of breast cancer growth by plant miR159. *Cell Res.* 2016;26(2):217–28. 10.1038/cr.2016.13 26794868 PMC4746606

[3] Liang H Zhang S Fu Z: Effective detection and quantification of *dietetically* absorbed plant microRNAs in human plasma. *J Nutr Biochem.* 2015;26(5):505–512. 10.1016/j.jnutbio.2014.12.002 25704478

[4] Baier SR Nguyen C Xie F: MicroRNAs Are Absorbed in Biologically Meaningful Amounts from Nutritionally Relevant Doses of Cow Milk and Affect Gene Expression in Peripheral Blood Mononuclear Cells, HEK-293 Kidney Cell Cultures, and Mouse Livers. *J Nutr.* 2014;144(10):1495–500. 10.3945/jn.114.196436 25122645 PMC4162473

[5] Snow JW Hale AE Isaacs SK: Ineffective delivery of diet-derived microRNAs to recipient animal organisms. *RNA Biol.* 2013;10(7):1107–1116. 10.4161/rna.24909 23669076 PMC3849158

[6] Witwer KW McAlexander MA Queen SE: Real-time quantitative PCR and droplet digital PCR for plant miRNAs in mammalian blood provide little evidence for general uptake of dietary miRNAs: Limited evidence for general uptake of dietary plant xenomiRs. *RNA Biol.* 2013;10(7):1080–1086. 10.4161/rna.25246 23770773 PMC3849155

[7] Dickinson B Zhang Y Petrick JS: Lack of detectable oral bioavailability of plant microRNAs after feeding in mice. *Nat Biotechnol.* 2013;31(11):965–967. 10.1038/nbt.2737 24213763

[8] Tosar JP Rovira C Naya H: Mining of public sequencing databases supports a non-dietary origin for putative foreign miRNAs: underestimated effects of contamination in NGS. *RNA.* 2014;20(6):754–757. 10.1261/rna.044263.114 24729469 PMC4024629

[9] Micó V Martín R Lasunción MA: Unsuccessful Detection of Plant MicroRNAs in Beer, Extra Virgin Olive Oil and Human Plasma After an Acute Ingestion of Extra Virgin Olive Oil. *Plant Foods Hum Nutr.* 2016;71(1):102–8. 10.1007/s11130-016-0534-9 26872816

[10] Auerbach A Vyas G Li A: Uptake of dietary milk miRNAs by adult humans: a validation study [version 1; referees: 3 approved]. *F1000Res.* 2016;5:721. 10.12688/f1000research.8548.1 27158459 PMC4857747

[11] Witwer KW: Contamination or artifacts may explain reports of plant miRNAs in humans. *J Nutr Biochem.* 2015;26(12):1685. 10.1016/j.jnutbio.2015.09.004 26456566

[12] Lusk RW: Diverse and widespread contamination evident in the unmapped depths of high throughput sequencing data. *PLoS One.* 2014;9(10):e110808. 10.1371/journal.pone.0110808 25354084 PMC4213012

[13] Kang W Bang-Berthelsen CH Holm A: Survey of 800+ data sets from human tissue and body fluid reveals xenomiRs are likely artifacts. *RNA.* 2017;23(4):433–445. 10.1261/rna.059725.116 28062594 PMC5340907

[14] Zhang Y Wiggins BE Lawrence C: Analysis of plant-derived miRNAs in animal small RNA datasets. *BMC Genomics.* 2012;13:381. 10.1186/1471-2164-13-381 22873950 PMC3462722

[15] Bağcı C Allmer J: One Step Forward, Two Steps Back; Xeno-MicroRNAs Reported in Breast Milk Are Artifacts. *PLoS One.* 2016;11(11):e0145065. 10.1371/journal.pone.0145065 26824347 PMC4732600

[16] Witwer KW Hirschi KD: Transfer and functional consequences of dietary microRNAs in vertebrates: concepts in search of corroboration: negative results challenge the hypothesis that dietary xenomiRs cross the gut and regulate genes in ingesting vertebrates, but important questions persist. *Bioessays.* 2014;36(4):394–406. 10.1002/bies.201300150 24436255 PMC4109825

[17] Zheng LL Deng KW Deng AC: Exo-miRExplorer: A Comprehensive Resource for Exploring and Comparatively Analyzing Exogenous MicroRNAs. *Front Microbiol.* 2017;8:126. 10.3389/fmicb.2017.00126 28203233 PMC5285362

[18] Liu YC Chen WL Kung WH: Plant miRNAs found in human circulating system provide evidences of cross kingdom RNAi. *BMC Genomics.* 2017;18(Suppl 2):112. 10.1186/s12864-017-3502-3 28361700 PMC5374554

[19] Ninomiya S Kawano M Abe T: Potential small guide RNAs for tRNase ZL from human plasma, peripheral blood mononuclear cells, and cultured cell lines. *PLoS One.* 2015;10(3):e0118631. 10.1371/journal.pone.0118631 25730316 PMC4346264

[20] Yuan T Huang X Woodcock M: Plasma extracellular RNA profiles in healthy and cancer patients. *Sci Rep.* 2016;6: 19413. 10.1038/srep19413 26786760 PMC4726401

[21] Zhang Z Yu J Li D: PMRD: plant microRNA database. *Nucleic Acids Res.* 2010;38(Database issue):D806–D813. 10.1093/nar/gkp818 19808935 PMC2808885

[22] Kozomara A Griffiths-Jones S: miRBase: annotating high confidence microRNAs using deep sequencing data. *Nucleic Acids Res.* 2014;42(Database issue):D68–73. 10.1093/nar/gkt1181 24275495 PMC3965103

[23] Jacobsen A Krogh A Kauppinen S: miRMaid: a unified programming interface for microRNA data resources. *BMC Bioinformatics.* 2010;11:29. 10.1186/1471-2105-11-29 20074352 PMC2831003

[24] Szczesniak MW Makalowska I: miRNEST 2.0: a database of plant and animal microRNAs. *Nucleic Acids Res.* 2014;42(Database issue):D74–D77. 10.1093/nar/gkt1156 24243848 PMC3965105

[25] Zhou Z Li X Liu J: Honeysuckle-encoded atypical microRNA2911 directly targets influenza A viruses. *Cell Res.* 2015;25(1):39–49. 10.1038/cr.2014.130 25287280 PMC4650580

[26] Yang J Hotz T Broadnax L: Anomalous uptake and circulatory characteristics of the plant-based small RNA MIR2911. *Sci Rep.* 2016;6: 26834. 10.1038/srep26834 27251858 PMC4890004

[27] Yang J Kongchan N Primo Planta C: The atypical genesis and bioavailability of the plant-based small RNA MIR2911: bulking up while breaking down. *Mol Nutr Food Res.* 2017;61(9): 1600974. 10.1002/mnfr.201600974 28319645 PMC5583006

[28] Griffiths-Jones S Grocock RJ van Dongen S: miRBase: microRNA sequences, targets and gene nomenclature. *Nucleic Acids Res.* 2006;34(Database issue):D140–4. 10.1093/nar/gkj112 16381832 PMC1347474

