# Supplementary material for: Alternative miRNAs? Human sequences misidentified as plant miRNAs in plant studies and in human plasma
Source: F1000Res. 2018 Feb 28;7:244. [Version 1] doi: 10.12688/f1000research.14060.1 (PMC5904727; doi:10.12688/f1000research.14060.1)
Supplement: Peer review history

# Peer review: Referee response for version 1

**DOI:** 10.5256/f1000research.15294.r31319

**Published:** 2018-04-13

**Authors:** Claus H. Bang-Berthelsen

**Affiliations:** 1 Microbial Biotechnology and Biorefining, National Food Institute, Technical University of Denmark, Kongens Lyngby, Denmark

K. W. Witwer presents some interesting findings in his brief report: Alternative miRNAs? Human sequences misidentified as plant miRNAs in plant studies and in human plasma. I consider the brief report scientifically good as well as well written.

 Additionally, I would like to recognize the effort made by K. W. Witwer on examination of the methodology and critical reviewing a recent publication by Liu and colleagues in 2017 ^1^ *.* Here Liu et al. report plant cross kingdom RNAi in two human sRNA plasma sequencing studies. Here Witwer identify a number of methodology issues in the Liu et al. paper by reanalyzing the public available data that were used in their paper. Witwer concludes that the most likely explanation for the Liu et al. reported plant RNA results, detected in the human plasma datasets, are traces of human degradome fragments and not of plant origin.

 The central conclusion in the manuscript are line with the findings that Kang et al. 2017 ^2^ reported based on a massive analysis of public available sRNA datasets comprising 10 billion sequencing reads. All in all supporting the null hypothesis that presence of xenomiRs are technical/contamination artifacts.

 Based on the overall impression of the paper it is scientifically sound and I can fully support the conclusions.

I have read this submission. I believe that I have an appropriate level of expertise to confirm that it is of an acceptable scientific standard.

## References

[1] LiuYChenWKungWHuangH: Plant miRNAs found in human circulating system provide evidences of cross kingdom RNAi. *BMC Genomics*.2017;18(S2) : 10.1186/s12864-017-3502-3 10.1186/s12864-017-3502-3 28361700PMC5374554

[2] KangWBang-BerthelsenCHHolmAHoubenAJMüllerAHThymannTPociotFEstivillXFriedländerMR: Survey of 800+ data sets from human tissue and body fluid reveals xenomiRs are likely artifacts. *RNA*.23(4) : 10.1261/rna.059725.116 433-445 10.1261/rna.059725.116 28062594PMC5340907

# Peer review: Referee response for version 1

**DOI:** 10.5256/f1000research.15294.r31318

**Published:** 2018-03-08

**Authors:** Jens Allmer

**Affiliations:** 1 Applied Bioinformatics, Bioscience, Wageningen University & Research , Wageningen, Netherlands

The article ‘Alternative miRNAs? Human sequences misidentified as plant miRNAs in plant studies and in human plasma’ by Kenneth W. Witwer addresses the recurring claim of plant xeno-microRNAs found in human plasma. Such xenomiRs are allegedly ingested and enriched in human; and this time Liu et al. claim evidence for cross kingdom RNAi via this route. Thus far, similar claims could not stand up against thorough validation as, for example, we (Bagci and Allmer, 2016 ^1^) and Kenneth W. Witwer (Auerbach et al. 2016 ^2^) have shown before.

 Here, Witwer has a closer look at the article ‘Plant miRNAs found in human circulating system provide evidence of cross kingdom RNAi’ by Liu et al. 2017 ^3^. Liu et al. 2017 claim that the plant miRNAs are not contamination, but only checked whether they contain sequencing adapters. Other sources of contamination were not considered. Additionally, Liu et al. 2017 reported to have used BLAST to check the plant miRNAs they found against the human genome without any similarities. Conversely, Witwer was able to find full-length identical alignments for these sequences in the human genome. For example, peu-MIR2910 is claimed by Liu et al to be conserved within comestible plants but a quick check on miRBase reveals that it is dead entry and not considered a miRNA anymore ( http://www.mirbase.org/cgi-bin/mirna_entry.pl?acc=MI0012633). Witwer correctly finds the mature sequence for this miRNA (UAGUUGGUGGAGCGAUUUGUC) in human while Liu et al. used the precursor sequence (UAGUUGGUGG AGCGAUUUGU CUGGUUAAUU CCGUUAACGA ACGAGACCUC AGCCUGCUA) for their search. I repeated the latter and found that the first 50 nucleotides are identical with several human RNAs (e.g.: NR_046235) which supports Witwer’s findings. It is of note, that PMRD is still not reachable and that the sequences I used were provided by Liu et al. in their commentary on Witwer’s article in biorxiv ^4^.

 Witwer points out that mapping results were not normalized which is supported by the original manuscript by Liu et al. However, such normalization is of essence especially when the number of observations is very low (spurious even?).

 I fully support the conclusions Kenneth W. Witwer draws in this article.

I have read this submission. I believe that I have an appropriate level of expertise to confirm that it is of an acceptable scientific standard.

## References

[1] BağcıCAllmerJ: One Step Forward, Two Steps Back; Xeno-MicroRNAs Reported in Breast Milk Are Artifacts. *PLoS One*.2016;11(1) : 10.1371/journal.pone.0145065 e0145065 10.1371/journal.pone.0145065 26824347PMC4732600

[2] AuerbachAVyasGLiAHalushkaMWitwerK: Uptake of dietary milk miRNAs by adult humans: a validation study. *F1000Res*.2016;5: 10.12688/f1000research.8548.1 721 10.12688/f1000research.8548.1 27158459PMC4857747

[3] LiuYChenWKungWHuangH: Plant miRNAs found in human circulating system provide evidences of cross kingdom RNAi. *BMC Genomics*.2017;18(S2) : 10.1186/s12864-017-3502-3 10.1186/s12864-017-3502-3 28361700PMC5374554

[4] WitwerKW: Alternative miRNAs: Human Sequences Misidentified As Plant miRNAs In Plant Studies And In Human Plasma. *bioRxiv*.2017;10.12688/f1000research.14060.1PMC590472729744036
